# Clinical Performance of the Reverse Transcription-Loop-Mediated Isothermal Amplification Assay for the Diagnosis of COVID-19 in a Thai Community Hospital at the Thailand-Myanmar Border

**DOI:** 10.7759/cureus.54447

**Published:** 2024-02-19

**Authors:** Thanee Wongchai, Nuttagarn Chuenchom, Wiphat Klayut, Benjawan Phetsuksiri, Payu Bhakdeenuan, Supranee Bunchoo, Sopa Srisungngam, Janisara Rudeeaneksin

**Affiliations:** 1 Infectious Diseases, Mae Sot Hospital, Ministry of Public Health, Tak, THA; 2 Hospital Medicine, Mae Sot Hospital, Ministry of Public Health, Tak, THA; 3 Infectious Diseases, National Institute of Health, Department of Medical Sciences, Ministry of Public Health, Nonthaburi, THA

**Keywords:** hospital, rrt-pcr, rt-lamp, sars-cov-2, covid-19

## Abstract

Introduction: Coronavirus disease 2019 (COVID-19) continues to be a global health threat and is a public health issue in Thailand and other countries. The extensive cross-border between Thailand and Myanmar is considered to be at a potentially high risk for COVID-19 distribution in this region. In this instance, simple and cost-effective tests for rapid and early detection of COVID-19 would be useful for effective patient management and control of the disease.

Methods: This study was conducted at Mae Sot Hospital on the border of Thailand-Myanmar to evaluate the diagnostic performance of a simple colorimetric reverse transcription-loop-mediated isothermal amplification (RT-LAMP) assay developed recently for the rapid detection of SARS-CoV-2. Nasopharyngeal specimens were routinely collected and processed through automated nucleic acid extraction followed by real-time reverse transcription-polymerase chain reaction (rRT-PCR) using the Molaccu^®^ COVID-19 Detection Kit. The RT-LAMP assay was further performed on remnant RNA samples, and the visual results were compared to those of rRT-PCR as a reference.

Results: Of the 727 samples tested, the RT-LAMP assay could detect 322 out of 374 samples positive for SARS-CoV-2 by rRT-PCR with 100% (n = 353/353) negative agreement. The comparative analysis demonstrated the overall accuracy, sensitivity, specificity, positive predictive value, and negative predictive value of the RT-LAMP at 92.85% (n = 675/727, 95% CI: 90.73-94.61), 86.10% (n = 322/374, 95% CI: 82.17-89.44), 100% (n = 353/353, 95% CI: 98.96-100), 100% (n = 322/322, 95% CI: 98.86-100), and 87.16% (n = 353/405, 95% CI: 84.06-89.73), respectively.

Conclusion: This RT-LAMP assay showed good diagnostic performance in the hospital setting. It can increase laboratory capacity for rapid SARS-CoV-2 testing and has the potential for use as an alternative or a backup assay at the point of need, especially where alternatives are unavailable for any reason, such as a decline in COVID-19 cases.

## Introduction

Coronavirus disease 2019, or COVID-19, an emerging respiratory infection caused by severe acute respiratory syndrome coronavirus 2 (SARS-CoV-2), remains a global health threat [1]. The disease is prevalent and continues to cause health issues in many regions. COVID-19 may disseminate more quickly as a result of frequent cross-border travel and low immunity. Tak Province, located in western Thailand, spans approximately 500 km along the border between Thailand and Myanmar. There is a significant number of border crossings, particularly in Tak’s Mae Sot District, involving Thais working temporarily in Myanmar and Burmese entering for employment, essential services, and healthcare. Mae Sot is the port of legal entry for travellers from Myanmar on the western border. Apart from this, a number of migrants illegally cross the Moei River, a small body of water that serves as a natural boundary between the two countries. The massive movement of Myanmar migrants and Thai people across the border increases the risk of potential infectious diseases, including COVID-19. There were approximately 150,000-300,000 Myanmar migrants who worked in the Mae Sot and its neighboring districts before the COVID-19 pandemic. This is a large number population, and among them, 40% worked in the garment business, another 40% were in agriculture, and 20% were in domestic labour and services [2]. Migration has significantly increased since the COVID-19 pandemic and the military coup in Myanmar in 2021 [2]. Meanwhile, the increasing numbers of illegal Myanmar migrants including refugees are undocumented or the data is not available. This factor contributed to the risk environment for COVID-19 distribution in this area.

The fast and accurate detection of SARS-CoV-2 infection is important for effective patient management and control of COVID-19. Simple and reliable diagnostics can increase access to testing and improve early case detection. The gold standard for confirming the clinical diagnosis of COVID-19 is real-time reverse transcription-polymerase chain reaction (rRT-PCR) due to its high sensitivity and specificity [3-4]. Although rRT-PCR is very accurate and can display results in a real-time manner, this testing is complex, expensive, and requires well-trained laboratory personnel. Its uses are, therefore, restricted to large, centralized, and well-equipped laboratories [5-6]. Concurrently, less expensive SARS-CoV-2 antigen tests and other simple devices for quick COVID-19 detection are more accessible and are being used more frequently as point-of-care (POC) diagnostics or at-home testing [7-8]. However, the main drawback of antigen tests is that they are less sensitive than rRT-PCR [9]. According to the recent meta-analysis data, immunochromatographic antigen tests for SARS-CoV-2 have a pooled sensitivity and specificity of roughly 68.4% and 99.4%, respectively [10]. In the best tests, the limit of detection regarding viral copies per millilitre of samples was about two to three times higher than that of rRT-PCR tests [9]. In this context, the simple and cost-effective molecular test would provide an alternative for efficient COVID-19 surveillance and control.

Loop-mediated isothermal amplification (LAMP) is a simple, fast, and low-cost molecular test through nucleic acid amplification under isothermal conditions [11]. Unlike PCR, the reaction requires strand displacement polymerase and four to six primers to amplify specific DNA sequences at a constant temperature [12]. Reverse transcription-LAMP (RT-LAMP) is added to detect the viral RNA [13]. The RT-LAMP procedure can be varied and is simply amendable while maintaining an acceptable level of sensitivity and specificity for the rapid detection of specific sequences within a shorter period than rRT-PCR. The colorimetric RT-LAMP assays allow for a simple readout of the amplification results by naked-eye visualization of the color change, providing an alternative for its application in different settings, including resource-limited laboratories [8,14]. Test sensitivity is varied according to the assay specification, sample type, and sample processing [14-15]. Recently, we developed and evaluated the performance of a simple colorimetric RT-LAMP, which exhibited good sensitivity and specificity for the rapid detection of SARS-CoV-2 in Thailand [16]. The primers used target highly conserved regions in an attempt to detect various kinds of known variants. Given that COVID-19 at the country’s borders may pose a serious health risk and Mae Sot Hospital serves both nationalities, we sought to ascertain the potential use and assess the diagnostic performance of our development in this setting. The data from this work can demonstrate the diagnostic performance of the test and serve as a basis for considering its use at Thailand’s borders or elsewhere.

## Materials and methods

Setting and study design

This cross-sectional study was conducted at Mae Sot General Hospital, a community hospital with 365 inpatient beds during 2021-2022. The hospital is located in the Mae Sot district of Tak province, Western Thailand, which shares a border with Myanmar. Nasopharyngeal swab (NPS) samples obtained through routine patient care for COVID-19 were used to evaluate the diagnostic performance of the recently developed RT-LAMP assay for the rapid detection of COVID-19 in comparison to standard rRT-PCR testing. All procedures performed in this hospital-based study were in accordance with ethical standards, and the study protocol was reviewed and approved by the Ethics Committee of Mae Sot Hospital, Ministry of Public Health (MoPH), Thailand (MSHP 24/2564). As RT-LAMP results were intended for research purposes, only the results of rRT-PCR were utilized for patient reporting.

Sample collection and handling

NPS specimens were collected from individuals clinically suspected of having COVID-19 and kept in a 2-ml viral transport medium (VTM). The collected samples in VTM-containing tubes were then kept in safety containers at 4°C, delivered to the laboratory, and tested by routine rRT-PCR in the diagnostic laboratory of Mae Sot Hospital.

The routine NPS specimens were selected for the study based on the inclusion and exclusion criteria. The inclusion criteria included suspected COVID-19 patients from both sexes aged over 18 years and with rRT-PCR testing results for detecting SARS-CoV-2 in the NPS specimens. The exclusion criteria were no rRT-PCR testing results or inconclusive rRT-PCR detection for SARS-CoV-2 in the NPS samples. The sample size was estimated based on an infinite population proportion using the following formula: n = (Z-score)^2^ * (*Se*) * (1-*Se*)/(d^2^ * *P*), where n = sample size; Z-score = the critical value and the standard value for the corresponding level of confidence (based on the confidence level; Z-score = 1.96 for confidence level 95%); *Se* = expected sensitivity of the test (RT-LAMP); d = margin of error; *P* = disease prevalence (COVID-19).

In this study, the proportion of SARS-CoV-2 infection among the studied population was estimated to be around 25%. The confidence level was taken as 95% and the margin of error as 5%. The expected sensitivity of the SARS-CoV-2 RT-LAMP was considered to be 85%. The sample size was then calculated. Patient samples were consecutively collected and registered until the sample size limit was achieved. The tests (rRT-PCR and RT-LAMP) were carried out by different investigators, and subsequently, the results were compared and analyzed.

Sample processing and analysis

RNA Extraction and Real-Time RT-PCR

Clinical specimens submitted for routine rRT-PCR testing for SARS-CoV-2 were extracted by automated nucleic acid extraction. Briefly, the total RNA was extracted from 200 µl of the NPS-suspended VTM using magnetic beads, with an automatic nucleic acid extractor (EXM3000; Zybio Inc., China) following the manufacturer’s instructions. The RNA was eluted in 200 µl of the elution buffer provided with the kit. The resulting RNA was used as RNA templates for detecting SARS-CoV-2 by rRT-PCR and RT-LAMP assays, respectively.

rRT-PCR Reference

A rRT-PCR assay of NPS samples was conducted as part of routine testing for COVID-19 diagnosis and used as the reference method for the evaluation study. The Molaccu® COVID-19 RT-PCR Detection Kit (Zybio Inc.), approved and regulated for in-country COVID-19 diagnosis by the Thailand Food and Drug Administration (FDA), was employed according to the manufacturer’s instructions. This assay targets RNA-dependent RNA polymerase (*RdRp*), nucleocapsid (*N*), SARS-CoV-2, and envelop (*E*) genes (Sarbecovirus) specific to SARS-CoV-2. According to testing recommendations, samples were considered positive for SARS-CoV-2 when the amplification cycle threshold (Ct) value was ≤40, at least two targets were detected, and the amplification curves exhibited a typical S shape. The data for the test’s limit of detection (LoD; 200 antigen units), accuracy (99.53%), clinical sensitivity (99.08%), and clinical specificity (100%) can be found online (https://covid-19-diagnostics.jrc.ec.europa.eu/devices/detail/2219). The hospital performed the rRT-PCR and carried out the reactions using the the CFX96 real-time PCR Detection System (Bio-Rad, Hercules, CA, USA).

RT-LAMP Assay

The newly developed RT-LAMP assay targeting the SARS-CoV-2 *orf8 *was carried out as described previously [16]. A primer set containing an outer forward primer (F3), outer backward primer (B3), forward inner primer (FIP), backward inner primer (BIP), loop forward primer (LF) and loop backward primer (LB) was used, as listed in Table 1. All primers were specific to SARS-CoV-2 based on a high sequence homology against SARS-CoV-2 with different lineages and variants, and a low sequence similarity with other microorganisms [16]. Prior to testing, the 10X LAMP primer mixture was prepared in nuclease-free water and stored at -20°C until use. In each RT-LAMP reaction, the final concentrations of primers were 0.2 μM F3/B3, 1.6 μM FIP/BIP, and 0.4 μM LF/LB, respectively.

**Table 1:** RT-LAMP primers targeting the SARS-CoV-2 orf8 used in this study RT-LAMP: reverse transcription-loop-mediated isothermal amplification; SARS-CoV-2: severe acute respiratory syndrome coronavirus 2

Primers	Nucleotide sequence (5’ to 3’)
F3-ORF8	TGGTATATTAGAGTAGGAGCTAGA	
B3-ORF8	AAACAACACGAACGTCATG	
FIP ORF8 (F2+F1c)	TCGATGTACTGAATGGGTGATTTAGTCAGCACCTTTAATTGAATTGTG	
BIP-ORF8 (B1c+B2)	AATTGCCAGGAACCTAAATTGGGCTCTAAAAAGTCTTCATAGAACGA	
LoopF-ORF8	AACCAGCCTCATCCACG	
LoopB-ORF8	AGTCTTGTAGTGCGTTGT	

The RT-LAMP reaction was set up in a total volume of 25 μl using the WarmStart® Colorimetric LAMP Master Mix (New England Biolabs, USA). Firstly, a reaction mix was prepared in accordance with the number of samples examined plus the control reactions. Each test reaction contained a volume of 20 µl reaction mix consisting of 12.5 µl WarmStart® Colorimetric LAMP Master Mix, 5 μl nuclease-free water, and 2.5 μl 10X primer mix at the concentrations previously described. The reaction mix was distributed into 0.2 mL PCR tubes, with each 5 μl of the RNA sample added immediately just before starting the reaction, followed by mixing and brief spinning down. After being incubated for amplification at 63°C for 60 minutes in a water bath or a heating block, the reaction tubes were taken out to stop the reaction on ice. The end-point results were examined directly by visual observation for the color shift and simply captured by a mobile phone camera. The transition from pink to yellow signified a positive result. For quality control, positive and negative controls were included in all running assays. For validated results, the positive control reactions should turn yellow, and the negative control reactions remain pink. The SARS-CoV-2 RNA extracted from SARS-CoV-2-positive samples was used as a positive control. The negative control was nuclease-free water with no SARS-CoV-2 RNA template.

Before testing the hospital samples, staff training and proficiency testing were performed to ensure the capability and accuracy of RT-LAMP testing. A set of samples positive by rRT-PCR for SARS-CoV-2, including negative and positive controls, were used in the proficiency testing.

Data and statistical analysis

The results of the RT-LAMP were compared to those of the rRT-PCR as a reference. The data were presented as frequencies or percentages with 95% confidence intervals (CIs). The accuracy, sensitivity, specificity, positive predictive value (PPV), and negative predictive value (NPV) of the RT-LAMP assay were determined by comparing the RT-LAMP results to those of the rRT-PCR testing and then computed. Stata/IC 16 for Windows (StataCorp, College Station, TX, USA) was used for statistical analyses.

## Results

Establishing the colorimetric RT-LAMP assay in a hospital laboratory

**Figure 1:**
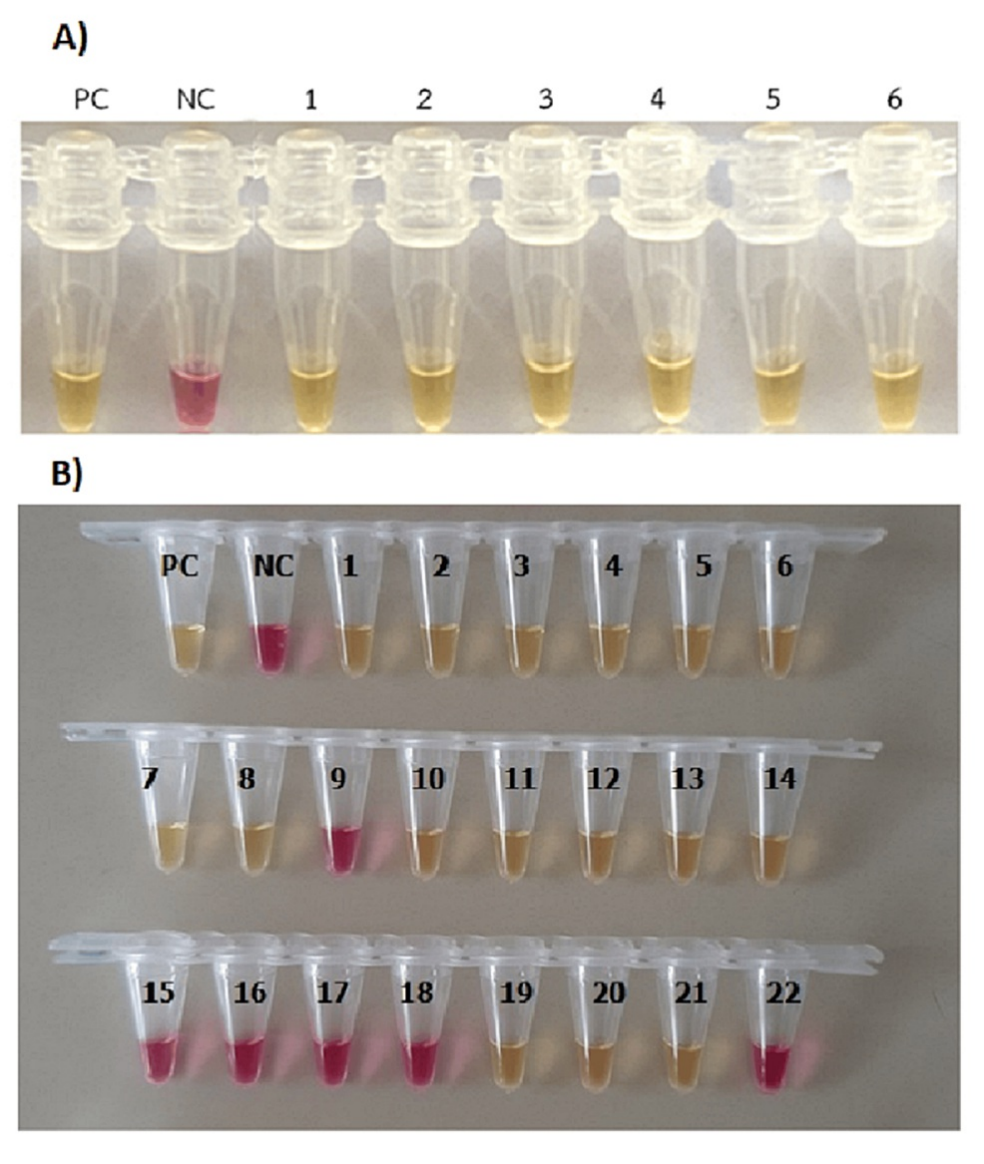
Results of colorimetric RT-LAMP assay using a new primer set targeting a specific portion of the orf8 for the detection of SARS-CoV-2 (A) RT-LAMP results in the proficiency test; 1-6 are proficiency test samples. (B) Representative RT-LAMP results in hospital samples; 1-22 are hospital samples. PC: positive control; NC: negative control; RT-LAMP: reverse transcription-loop-mediated isothermal amplification; SARS-CoV-2: severe acute respiratory syndrome coronavirus 2

The RT-LAMP assay could be set up in the hospital laboratory, where the reactions could be carried out and a color change from pink to yellow was displayed after amplification of the SARS-CoV-2 *orf8* at 63°C for 60 min (Figures 1A, 1B). Detection of the end-point results could be visualized for a color change by the naked eye, with the images simply recorded using a cell phone camera.

The results to verify the proficiency of the RT-LAMP testing were obtained after training the hospital laboratory personnel in the execution of the assays on-site at Mae Sot Hospital. Table 2 and Figure 1A show the proficiency testing results.

**Table 2:** Evaluation results of the RT-LAMP proficiency test for the detection of SARS-CoV-2 at Mae Sot Hospital RT-LAMP: reverse transcription-loop-mediated isothermal amplification; SARS-CoV-2: severe acute respiratory syndrome coronavirus 2; Ct: cycle threshold; IC: internal control; rRT-PCR: real-time reverse transcription-polymerase chain reaction

Samples	Ct values of positive RT-PCR results				RT-LAMP result
	*E* gene	*N* gene	*RdRp* gene	IC	
1	16.31	16.53	18.63	19.93	+
2	13.60	13.30	15.06	18.32	+
3	13.04	13.44	13.31	21.17	+
4	23.40	22.84	24.64	20.35	+
5	21.69	22.21	22.61	24.93	+
6	33.03	32.90	32.62	18.80	+

Clinical performance of the RT-LAMP assay in a hospital setting

A total of 727 surplus nasopharyngeal RNA samples with corresponding rRT-PCR Ct data were obtained anonymously for RT-LAMP testing. Of these, 374 samples were rRT-PCR positive, and the remaining 353 rRT-PCR negative for SARS-CoV-2 RNA. The RT-LAMP results from all tested samples were compared with those of rRT-PCR. Visualization of the RT-LAMP results revealed that 322 out of 374 samples (86.10%, 95% CI: 82.17-89.44) that tested positive by rRT-PCR were also positive by RT-LAMP. Additionally, all 353 samples (100%) that tested negative by rRT-PCR were negative by RT-LAMP (Table 3). A consistency of 100% negative agreement (n = 353) indicated satisfied specificity. In comparison to rRT-PCR, the overall diagnostic accuracy, sensitivity, specificity, including PPV and NPV of the RT-LAMP, were 92.85% (n = 675/727; 95% CI: 90.73-94.61), 86.10% (n = 322/374, 95% CI: 82.17-89.44), 100% (n = 353/353, 95% CI: 98.96-100), 100% (n = 322/322, 95% CI: 98.86-100), and 87.16% (n = 353/405, 95% CI: 84.06-89.73), respectively. A summary of the RT-LAMP results and clinical performance of the RT-LAMP assay in comparison to rRT-PCR detection is presented in Table 3.

**Table 3:** Comparative results of RT-LAMP and rRT-PCR based on Ct values for the detection of SARS-CoV-2 in a hospital setting RT-LAMP: reverse transcription-loop-mediated isothermal amplification; rRT-PCR: real-time reverse transcription-polymerase chain reaction; Ct: cycle threshold; SARS-CoV-2: severe acute respiratory syndrome coronavirus 2; n: sample number

Tested samples		rRT-PCR		Accuracy	Sensitivity	Specificity	PPV	NPV
(n = 727)		Positive	Negative	(95% CI)	(95% CI)	(95% CI)	(95% CI)	(95% CI)
RT-LAMP	Positive	322	0	92.85% (n = 675/727)	86.10% (n = 322/374)	100% (n = 353/353)	100% (n = 322/322)	87.16% (n = 353/405)
	Negative	52	353	(90.73-94.61%)	(82.17-89.44%)	(98.86-100%)	(98.96-100%)	(84.06-89.73%)

To further evaluate the clinical performance of the RT-LAMP assay, the detection results were also compared to those of rRT-PCR based on the Ct values of rRT-PCR positive samples. Table 4 summarizes the positive rate of the RT-LAMP assay in comparison to rRT-PCR based on Ct values targeting *N*, *RdRp*, and *E* genes.

**Table 4:** Comparison of the detection results and diagnostic performance between the RT-LAMP assay and rRT-PCR for detecting SARS-CoV-2 in clinical specimens RT-LAMP: reverse transcription-loop-mediated isothermal amplification, rRT-PCR: real-time reverse transcription-polymerase chain reaction, SARS-CoV-2: severe acute respiratory syndrome coronavirus 2, n: sample number, CI: confidence interval, PPV: positive predictive value, NPV: negative predictive value

		rRT-PCR positive samples based on detected Ct values (n = 374)					
Tested samples							
(n = 374)		≤25	>25-30	>30-35	>35		
RT-LAMP	Positive							239/239 (100%)	42/46 (91.30%)	22/48 (45.83%)	19/41 (46.34%)	N	Targets
	Negative	0	4/46 (8.70%)	26/48 (54.17%)	22/41 (53.66%)								
	Positive	236/236 (100%)	42/46 (91.30%)	26/45 (57.78%)	18/47 (38.30%)	RdRp							
							Negative	0	4/46 (8.70%)	19/45 (42.22%)	29/47 (61.70%)		
	Positive	242/243 (99.59%)	41/44 (93.18%)	25/54 (46.30%)	14/33 (42.42%)							E	
							Negative	1/243 (0.41%)	3/44 (6.82%)	29/54 (53.70%)	19/33 (57.58%)		

**Figure 2:**
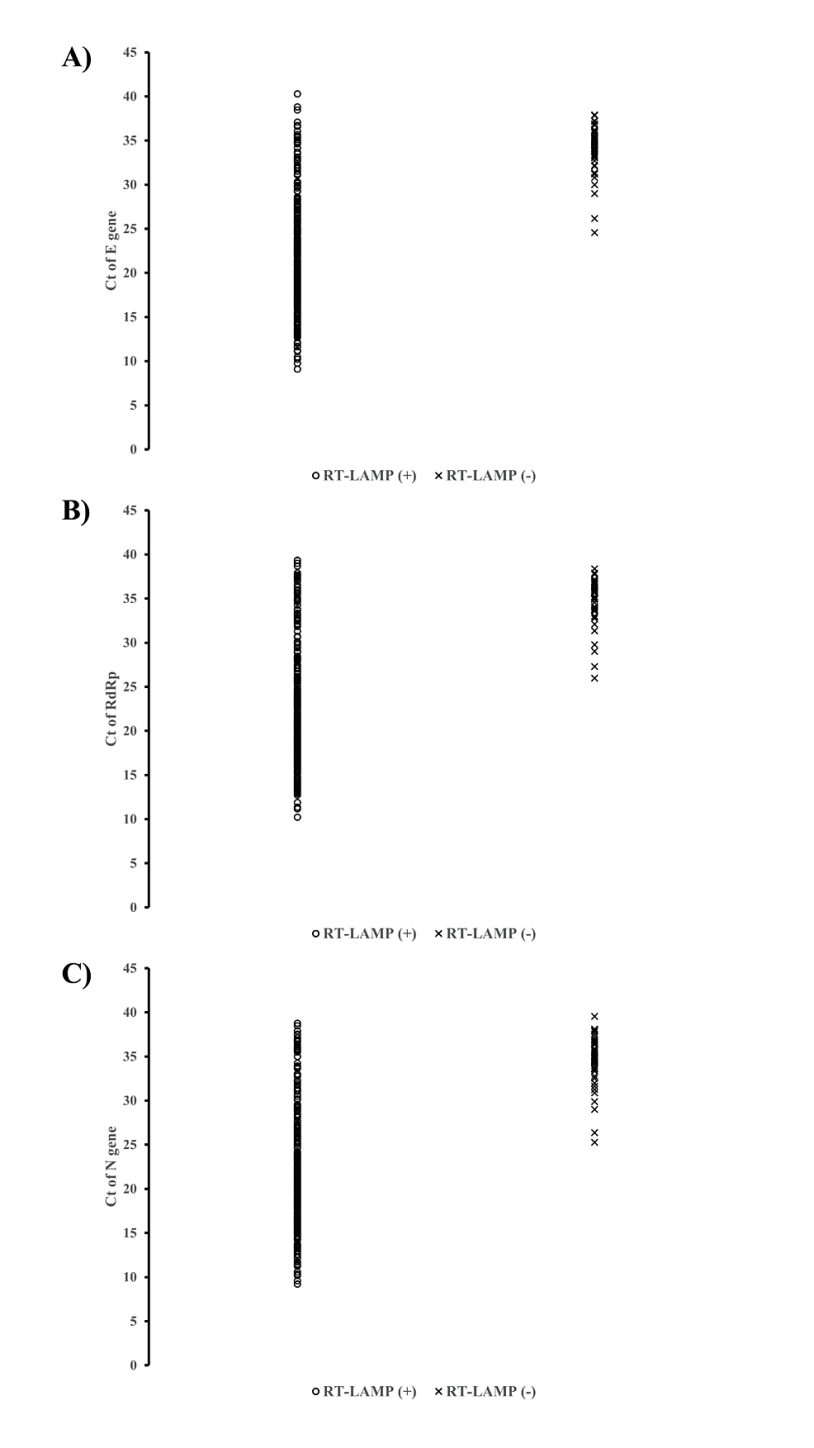
Distribution of RT-LAMP results in comparison to rRT-PCR detection based on Ct values The Ct values (rRT-PCR results) of 322 COVID-19-positive samples (y-axis) were compared to the RT-LAMP readout results (x-axis) taken after 60 min of incubation at 63°C: (A) Ct values of *E* gene, (B) Ct values of *RdRp* gene, (C) Ct values of *N* gene. RT-LAMP: reverse transcription-loop-mediated isothermal amplification; rRT-PCR: real-time reverse transcription-polymerase chain reaction; Ct: cycle threshold

The comparative results showed that the RT-LAMP demonstrated high sensitivity in the majority of rRT-PCR positive samples with Ct values at ≤30 cycles. Based on rRT-PCR Ct values at ≤25 cycles, the sensitivities of the RT-LAMP among samples that tested positive by rRT-PCR targeting the *N*, *RdRp*, and* E* genes were 100% (n = 239/239), 100% (n = 236/236), and 99.59% (n = 242/243), respectively (Table 4). In samples with Ct values at >25-30 cycles, the sensitivities of the RT-LAMP slightly declined to 91.30% (n = 42/46) to 93.18% (n = 41/44) compared to rRT-PCR positive results targeting *N* (n = 42/46; 91.30%), *RdRp* (n = 42/46; 91.30%), and *E* (n = 41/44; 93.18%) genes, respectively (Table 4). In samples with Ct values at >30-35 cycles, the sensitivities declined to 45.83% (n = 22/48) to 57.78% (n = 26/45). It should be noted that about 40% of positive samples with Ct values at >35 cycles could be detected by the RT-LAMP, corresponding to the sensitivity reduction to 46.34% (n = 19/41), 38.30% (n = 18/47), and 42.42% (n = 14/33) based on rRT-PCR detection targeting *N*, *RdRp* and *E* genes, respectively (Table 4). The highest sensitivity of the RT-LAMP assay ranged from 99.6% to 100% among samples with Ct values ≤25 cycles. The sensitivity was still high at >90% in samples with Ct values of >25-30 cycles but dropped to about 50% in samples with Ct values at >30 cycles. Table 4 shows the varying sensitivities of the RT-LAMP assay based on Ct values of positive rRT-PCR. Most of the samples observed to be positive by rRT-PCR (>60%) had Ct values of <25 cycles, demonstrating that 99% of these could be detected by the RT-LAMP. Overall, the diagnostic sensitivity of this RT-LAMP assay in the real-world study was relatively high (86.10%) compared to rRT-PCR. The distribution of the RT-LAMP positive and negative results in comparison to the rRT-PCR detection targeting *N*, *RdRp*, and *E* genes is presented in Table 4 and Figure 2, respectively.

## Discussion

This study examined the diagnostic performance of the RT-LAMP for the rapid detection of SARS-CoV-2 infection by comparing it with the rRT-PCR test in the real-world setting of a remote hospital. The RT-LAMP assay was evaluated to detect COVID-19 cases among suspected COVID-19 patients living in the Mae Sot district community at the border of Thailand-Myanmar. The study setting was considered to have a high exposure risk for COVID-19 infection considering numerous migrations, congregate living or working in poor ventilation, low rates of access to healthcare and low vaccination coverage against COVID-19. During the field evaluation, the prevalence of COVID-19 in the Mae Sot district was considered to be high (25%) based on the high positive detection rate of COVID-19 infection due to COVID-19 outbreaks. Mae Sot Hospital is a community hospital that has rRT-PCR facilities. In routine practice, clinical specimens for COVID-19 diagnosis were collected at the hospital (sample collecting sites or mobile sample collecting units) and then carried to the hospital laboratory. In many instances, samples were taken outside or at remote healthcare delivery sites and transported to adjunct central facilities or healthcare delivery centres with rRT-PCR instruments like Mae Sot hospital. The collection and handling of specimens from individuals suspected of having SARS-CoV-2 infection was performed with adequate personnel protective equipment (PPE) and infection control precautions in accordance with World Health Organization (WHO) interim guidance on COVID-19 specimen collection and transportation [17]. In addition, the specimen handling or transportation was performed by authorized personnel with training in safe handling practice and spill decontamination protocols. At the time of sample collection, different lineages and sublineages of SARS-CoV-2 were identified with the majority of Delta and various Omicron variants. In general, diagnostic tests cannot identify the specific SARS-CoV-2 variant causing infection. To genotype, viral sequencing or specialized multiplex PCR is needed. The information on SARS-CoV-2 lineages as described was obtained from the summaries of the country reports on SARS-CoV-2 lineages in Thailand’s population. Overall, the study demonstrated the potential for use and good performance of the recently developed RT-LAMP targeting the highly conserved regions of SARS-CoV-2 *orf8*. The data obtained can be used to support the decision to utilize RT-LAMP to detect SARS-CoV-2. It can be used as an alternative for the rapid detection of COVID-19 in border areas where humans and pathogens move across geographic boundaries critical for the potential spread or outbreak of COVID-19.

Likewise, rRT-PCR is the reference standard for diagnosing COVID-19 in Thailand. The government primarily facilitated reimbursement for each laboratory testing through the Universal Health Coverage processed by the National Health Security Office (NHSO), allowing hospitals to manage these funds independently. Eligible patients covered by NHSO support, therefore, do not have to pay for COVID-19-related healthcare services, such as SARS-CoV-2 rRT-PCR testing. Additional financing sources came from government healthcare programs and individual health insurance. Otherwise, healthcare services and testing for COVID-19 are paid independently. Partial support for instruments or consumable supplies also came from non-government organizations, international supports, and research funding including special programs or collaborations. SARS-CoV-2 rRT-PCR testing is conducted on specimens in accordance with physicians’ decisions following the guidance for the diagnosis and reporting of COVID-19 [17]. Although the RT-LAMP assay is not a reference standard for COVID-19 diagnosis, the technique was approved as an alternative molecular test that can be used for in-country COVID-19 diagnosis. The government does not provide specific support for instruments or consumables at healthcare facilities for COVID-19 testing. However, it is feasible to set up the RT-LAMP assay in the laboratory even in rural areas by using simple equipment like a small heating block or a water bath, which are not expensive to purchase or are generally available in laboratories. According to its affordability, simplicity, accuracy, and low cost, RT-LAMP has potential application. It can be used in resource-limited settings where the rRT-PCR instrument is not available. The intended application and enabling policies including the availability of reasonable tests also influence how feasible its use is.

Similarly, it has been previously reported that the majority of specimens tested for SARS-CoV-2 were NPS [18]. In this study, we tested NPS specimens, and the results demonstrated an overall sensitivity of 86.10% (n = 322/374) and a specificity of 100% (n = 353/353) for the RT-LAMP. The rRT-PCR-negative samples for SARS-CoV-2 were included in the evaluation even though it was recognized that the minimal virus copies required for positive detection by the RT-LAMP test were higher than rRT-PCR. The evaluation demonstrated good specificity of the test based on no positivity among rRT-PCR-negative samples. Many factors affected the molecular detection of SARS-CoV-2. The sensitivity and accuracy of RT-LAMP assays also vary from test to test [19]. However, the selected tests for use must meet the necessary standards according to WHO’s target product profiles for COVID-19 diagnostics, which call for an acceptable level of sensitivity and specificity of at least 80% and 97%, respectively [20]. These can be taken into consideration as an alternative to rRT-PCR, especially when or where rRT-PCR tests are unavailable or the confirmation of antigen testing for COVID-19 is required [20]. According to our results, the overall sensitivity of this RT-LAMP in a hospital setting reached 80% compared to rRT-PCR detection. Therefore, its performance met the priority criteria according to WHO’s recommendations [21].

Based on rRT-PCR Ct values, the clinical performance of the RT-LAMP was evaluated in further detail. The RT-LAMP assay missed a positive detection in a small number of samples with Ct values at <30 cycles as determined by the rRT-PCR reference, with the highest sensitivity of RT-LAMP being around 99% in samples with Ct values at ≤25 cycles. Meanwhile, the RT-LAMP assay had a sensitivity drop of roughly 50% in samples testing positive for rRT-PCR with Ct values at >30 cycles, and a sensitivity reduction was frequently observed in samples with high Ct values or a low viral load. The diagnostic performance of the RT-LAMP in this study can be further compared with commercial RT-LAMP tests or other RT-LAMP developments. Previously, the clinical sensitivity, specificity, PPV, and NPV of the Loopamp SARS-CoV-2 Detection kit (Eiken Chemical, Tokyo, Japan) were reported to be 78.9%, 100%, 100%, and 55.6%, respectively [22]. Silva et al. reported the clinical evaluation of the RT-LAMP in a hospital setting with 95.83% accuracy, 88.89% sensitivity, and 98.55% specificity [23]. It was noted that other factors, such as specimens and specimen processing, could affect the clinical sensitivity of RT-LAMP [14-15,24]. For sample types, the RT-LAMP assay shared many considerations like rRT-PCR [8]. It can be used to test a variety of upper and lower respiratory specimens. NPS, mid-turbinate swabs, anterior nasal swabs, oropharyngeal swabs, and saliva are the commonly used specimens. Test sensitivity can be affected by specimen types according to different viral loads varied by tissue affinity for the virus [8]. Regarding specimen processing, the RT-LAMP test has the potential to be performed on crude samples to save cost and time; however, based on prior published studies, it has been suggested that RNA extraction improved the test’s sensitivity. A recent meta-analysis on the evaluation of the RT-LAMP assay for the rapid detection of SARS-CoV-2 reported that the pooled sensitivity of RT-LAMP in samples with RNA extraction was higher at 88% (86%-90%), compared to that in samples without RNA extraction or crude samples, at 50% (45%-55%) [19]. Different RNA extraction procedures also provide different quality and quantity of RNA yields, and hence, may affect the sensitivity of the test. In addition, the sensitivity and accuracy of tests can be affected by the reference standard used in the evaluation [25]. In comparison to other RT-LAMP developments, this RT-LAMP assay showed sensitivity similar to a previous meta-analysis, which reported that the overall sensitivity of RT-LAMP assays usually ranged from 75% to 100% compared to rRT-PCR [26].

Recently, our RT-LAMP assay was demonstrated to have an estimated LoD of fewer than 25 copies of SARS-CoV-2 RNA per reaction [16]. As expected, the good clinical performance of this assay was exhibited in a hospital setting. In addition, this study also demonstrated that a colorimetric RT-LAMP method could be established and integrated into routine facilities. Due to its simplicity, the RT-LAMP assay can be employed for the rapid detection of SARS-CoV-2 infection within healthcare facilities. In terms of test costs, the RT-LAMP was cheaper than the rRT-PCR. Our RT-LAMP reaction costs about USD 5-7 per test while the Zybio rRT-PCR test kit costs roughly USD 10-12. According to the diagnostic performance, the developed RT-LAMP is sufficiently sensitive and reliable for detecting SARS-CoV-2, indicating that it can be used, as intended, in a hospital setting.

A large number of positive samples were included in this work due to the spread of COVID-19 at the border during the study period. The study also provided a representative insight into the diagnostic performance of the in-house RT-LAMP assay in detecting the range of viral load present in patients’ samples. However, there were some limitations. We used excess RNA materials from a routine diagnostic procedure, and it was not feasible to evaluate the RT-LAMP method for the direct detection of SARS-CoV-2 in unprocessed or crude samples. In addition, we were unable to assess the potential of the RT-LAMP assay to detect SARS-CoV-2 in a variety of specimens. Avoiding interfering with routine procedures, heavy workloads and resource constraints also restricted the study. Despite these limitations, the assay demonstrated its possible value as an alternative or a backup assay for increased testing and reliable and timely detection of COVID-19 in Thailand.

## Conclusions

The RT-LAMP assay exhibited good diagnostic performance in a large sample tested on-site in a hospital setting, with an overall 86.10% sensitivity and 100% specificity for the rapid detection of SARS-CoV-2. The sensitivity of the RT-LAMP assay decreased as the Ct values of the rRT-PCR increased. The greatest sensitivity was about 99% in samples with Ct values at ≤25 cycles and approximately 91%-93% in samples with Ct values at >25-30 cycles. The study also highlights the importance of test evaluation in the intended clinical setting where the targeted population is at a high risk. The assay can increase the availability of SARS-CoV-2 testing and expand the testing capacity of laboratories.
